# First person – Piotr Soczewka

**DOI:** 10.1242/dmm.039016

**Published:** 2019-01-28

## Abstract

First Person is a series of interviews with the first authors of a selection of papers published in Disease Models & Mechanisms (DMM), helping early-career researchers promote themselves alongside their papers. Piotr Soczewka is first author on ‘Yeast-model-based study identified myosin- and calcium-dependent calmodulin signalling as a potential target for drug intervention in chorea-acanthocytosis’, published in DMM. Piotr is a PhD student in the lab of Prof. Treresa Żołądek at the Polish Academy of Sciences, Warsaw, Poland, investigating the use of yeast to study neurodegenerative diseases.


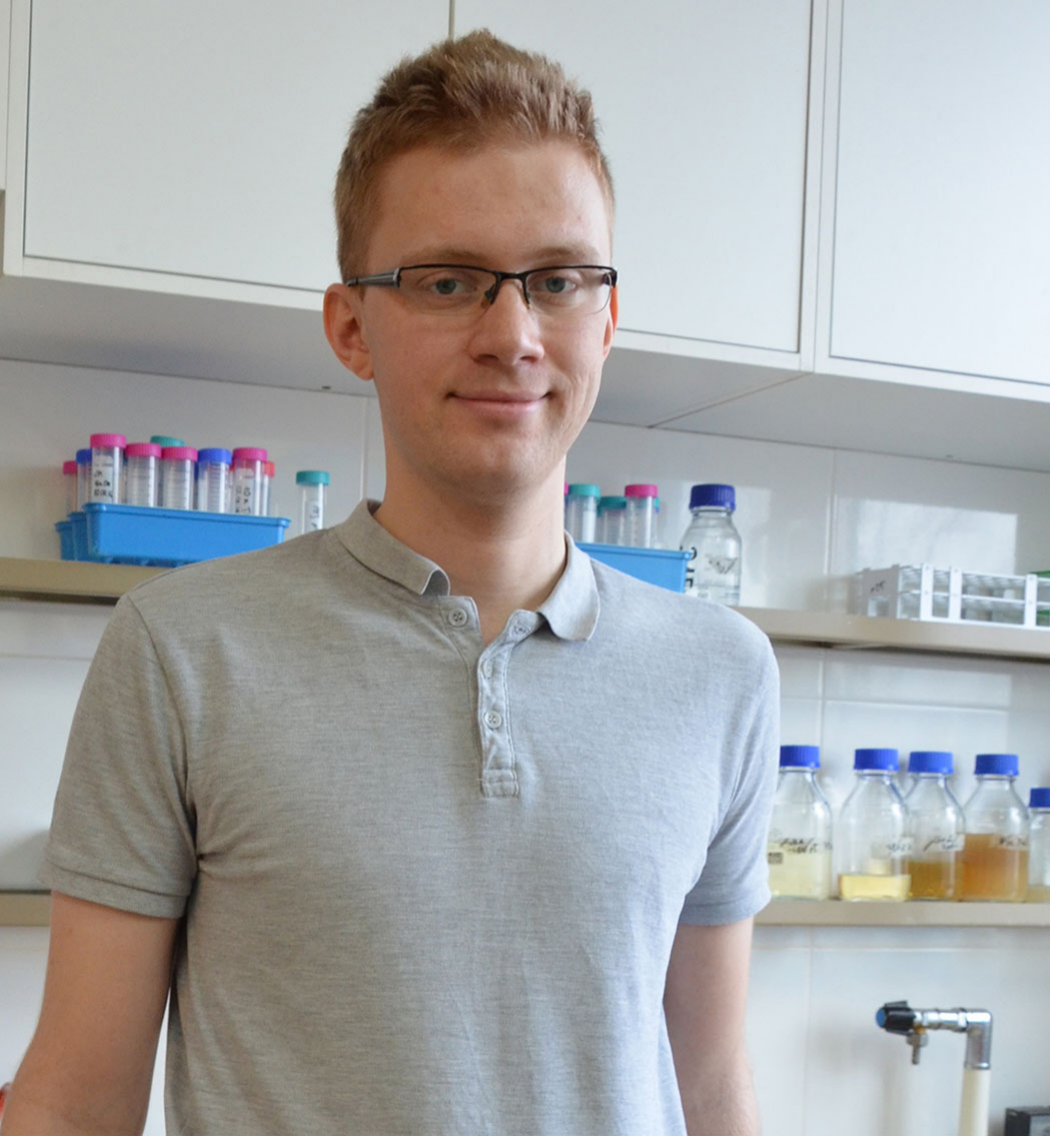


**Piotr Soczewka**

**How would you explain the main findings of your paper to non-scientific family and friends?**

No matter how ridiculous it may seem, I use yeast for studying neurodegenerative diseases. This approach is justified by the facts that human and yeast cells function in a similar way and many genes associated with human diseases have their corresponding gene in yeast. By genetic manipulations, one can introduce a mutation in yeast causing alterations on a cellular level, which reflect defects present in patients with a particular disease. In my research, I am focused on chorea-acanthocytosis (ChAc) – a rare neurodegenerative disorder caused by mutations in the *VPS13A* gene. To obtain a yeast ChAc model, we created a *vps13*Δ yeast strain, in which the *VPS13* gene was removed.

We noticed that the *vps13*Δ strain grows poorly, compared to the strain with intact *VPS13* gene, particularly in stress conditions. In our study, we used this observation to search for genetic factors that are able to restore the growth of the *vps13*Δ strain and, thus, to bypass defects caused by lack of the *VPS13* gene. We discovered that *vps13*Δ functioning was improved by the factors that diminish effects caused by increased calcium concentrations. Our findings point at calcium regulation as a potential therapeutic target in ChAc patients.

**What are the potential implications of these results for your field of research?**

Presented results are the starting point for follow-up studies on ChAc using higher organisms or cell lines as models. In the long term, they may contribute to establishing an effective therapy for ChAc patients. Also, we provide a phenotype of a trafficking and endocytic mutant, which could be used for genetic and chemical suppressor screens.

“Simplicity is at the same time the biggest advantage and the biggest drawback of the yeast model.”

**What are the main advantages and drawbacks of the model system you have used as it relates to the disease you are investigating?**

Simplicity is at the same time the biggest advantage and the biggest drawback of the yeast model. Work with this unicellular organism is undoubtedly much more convenient than with the higher eukaryotes. The experimental material grows by itself overnight and genetically engineered strains can be created within a few days. These features significantly speed up procedures and allow us to test more ideas and, thus, make research more enjoyable. On the other hand, disease modelling is limited only to the cellular level. Defects on tissues or at organ system levels are obviously impossible to study. Also, due to far evolutionary distance, obtained result are not directly transferable to humans. Findings should be confirmed on evolutionarily closer model organisms, such as mice.

**Describe what you think is the most significant challenge impacting your research at this time and how will this be addressed over the next 10 years?**

Recently, Vps13 proteins have been shown to act as lipid transfer proteins, and this suggests that impaired lipid distribution may be the cause of ChAc. However, the exact mechanisms of molecular pathology remains unknown. Advanced methods of lipidomics may be helpful in determining changes that trigger neurodegeneration in patients.

“I believe that one of the main reasons why young people are discouraged from pursuing an academic career is the lack of stable job (hence stable life) perspectives.”

**Figure:**
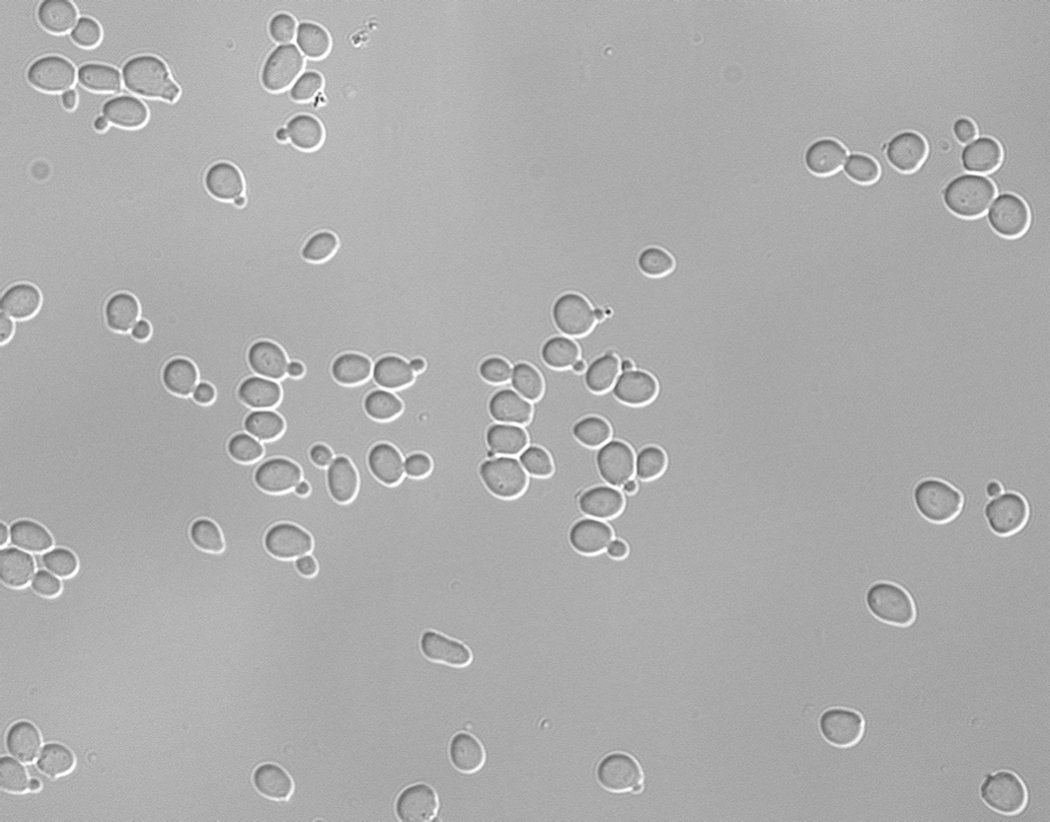
**Microscope photo of yeast cells.**

**What changes do you think could improve the professional lives of early-career scientists?**

I believe that one of the main reasons why young people are discouraged from pursuing an academic career is the lack of stable job (hence stable life) perspectives. Apart from making permanent positions more accessible, I think that training courses focused on practical skills should be organised for every PhD student, regardless of their PhD projects. This would increase the value of a graduate PhD in the job market and broaden future job possibilities.

**What's next for you?**

For now, I plan to pursue my current research with the yeast ChAc model and to complete my PhD. After that, I would like to apply for a postdoc position abroad, but I am also considering a science-related job in industry.
